# Meta-analysis of the role of zinc in coordinating absorption of mineral elements in wheat seedlings

**DOI:** 10.1186/s13007-021-00805-7

**Published:** 2021-10-12

**Authors:** Xiaolong Guo, Xiangyu Ma, Jialiang Zhang, Jinghuan Zhu, Tian Lu, Qifei Wang, Xiaoming Wang, Wei Hua, Shengbao Xu

**Affiliations:** 1 State Key Laboratory of Crop Stress Biology for Arid Areas, College of Agronomy, Northwest A&F University, Yangling, Xianyang, 712100 Shaanxi China; 2 Zhejiang Academy of Agricultural Sciences, Hangzhou, 310021 Zhejiang China

**Keywords:** Wheat, Zinc, Mineral elements absorption, New method, Cultivars

## Abstract

**Background:**

Zinc (Zn) is an important nutrient for human beings, which is also an essential micronutrient for crop growth. This study investigated the role of Zn in coordinating the mineral elements absorption in modern wheat (*Triticum aestivum* L*.*) cultivars with a new developed method.

**Results:**

A method was developed, and showed a robust capability to simultaneously investigate seven mineral elements uptake in wheat seedling. With this method, we found low Zn supply (<  1 μM) promoted the absorption of potassium (K), magnesium (Mg) and manganese (Mn) in wheat seedling, while high Zn supply (>  1 μM) significantly inhibited the absorption of these elements. Cultivars with the green genes (*Rht-B1b* and *Rht-D1b*) showed a higher uptake capability on ammonium (NH_4_^+^), and cultivars with *Rht-B1b* allele can uptake more phosphors (P), K, calcium (Ca), Mn and Zn compared to cultivars with *Rht-D1b*. Further analysis indicated higher uptake capability of NH_4_^+^ in cultivars contained *Rht*s was independent of Zn.

**Conclusion:**

The key role of Zn in coordinating for mineral elements absorption was identified in modern wheat cultivars, providing the reference for Zn application in wheat. Meanwhile, this study provides a robust method for quantifying the absorption of mineral elements, which may be adopted into the broadly investigations on the coordinated nutrients absorption of plant.

**Supplementary Information:**

The online version contains supplementary material available at 10.1186/s13007-021-00805-7.

## Background

To enhance the yield, chemical fertilizer has become the main source of mineral elements for all kinds of crops [1, 2]. While these inputs help to meet the demand for crop yields, then overuse of fertilizers also causes serious environmental damage, such as the greenhouse effect and water eutrophication [3]. Therefore, it is important to understand the mineral element absorption rules of crops for rational fertilization.

Zn is an essential nutrient element for plants and humans, which is widely involved in biochemical and physiological processes, including enzyme activity, cell division, photosynthesis, carbohydrate and lipid metabolism, detoxification of oxygen radicals and protein synthesis [4–7]. Zn deficiency is common in most crops, which causes leaf chlorosis, curling and wilting, and stunted and thin stems [8–10], and ultimately leads to low grain yields and poor grain quality [11, 12]. Foliar application of Zn can be absorbed through the cuticle and/or the stomates, and then transported to leaves, stems, reproductive tissues and grains, to rescue the symptom of Zn deficiency [13, 14]. Proper supply Zn can promote plant growth and development by enhancing photosynthesis and carbohydrate metabolism [15]. On the other side, excess Zn can lead to leaf necrosis [16], biomass decline [17], affect the absorption of other elements [18] and many adverse effects on crop growth and development [5, 19]. Previous studies focused on the effects of other elements on Zn uptake in crops [20–22]. However, the effect of Zn in regulating other mineral elements absorption is still largely unknown, which hinders our understanding of the rational use of Zn fertilizer in agricultural production.

The commonly application of dwarfing genes *Rht-B1b* and *Rht-D1b* significantly increases modern wheat yield and the high-input of nitrogen fertilizers is the key to achieve the maximum its yield potential [23–26]. Previous studies have shown that *Rhts* allele significantly altered iron (Fe), Zn, copper (Cu) and Mg content in wheat grains [24, 27], then the underlying mechanism still keeps in largely unknown.

At present, the research on nutrient elements absorption in crops mainly adopts tissue digestion method to determine the content of mineral elements in plant tissues [24, 28, 29]. However, this method is relatively tedious and also has the risk because of the strong acid application. Therefore, the aim of this study was to develop a new method for quantifying the absorption of mineral elements and identify the role of Zn in the coordinated absorption of mineral elements in wheat seedling.

## Results

### A new method for quantifying mineral elements uptake in wheat seedling

**Fig. 1:**
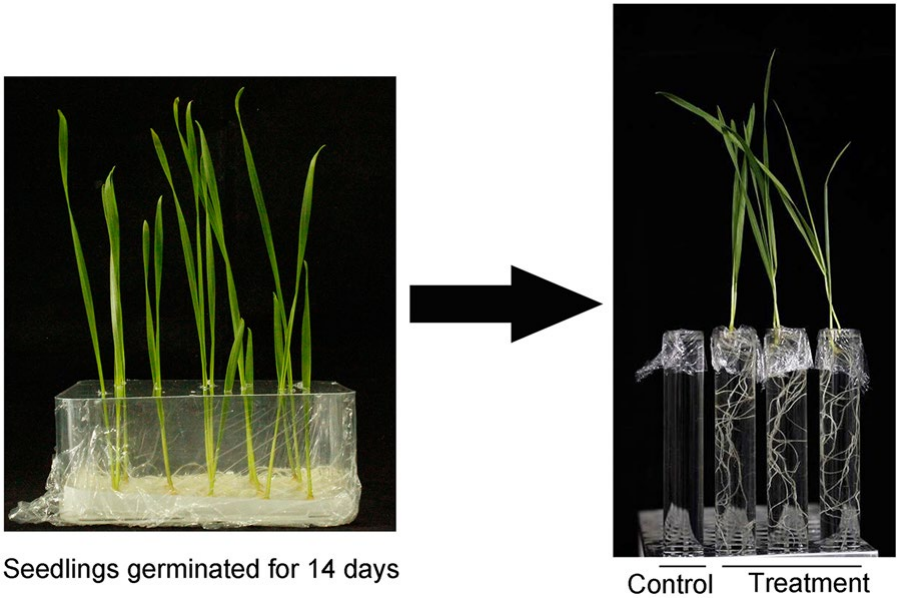
Scenes of tube nutrient solution treatment. Wheat seedlings that had been hydroponic for two weeks after germination were transferred to tubes containing nutrient solution for treatment. The test tube mouth is covered with a film to reduce the loss of water and nutrients. Nutrient solution in test tube without treatment of seedlings were used as control

To investigate the mineral elements uptake in wheat seedling, a nutrient solution quantification in the test tube was developed. Briefly, hydroponic wheat seedling (14 days after germination) was transferred to the tube containing 1/2 Hoagland nutrient solution with different treated time (Fig. 1), then quantifying the reduction of mineral elements in solution by atomic absorption spectrophotometer and continuous flow analyzer, to represent the uptake by the wheat seedling.

**Fig. 2:**
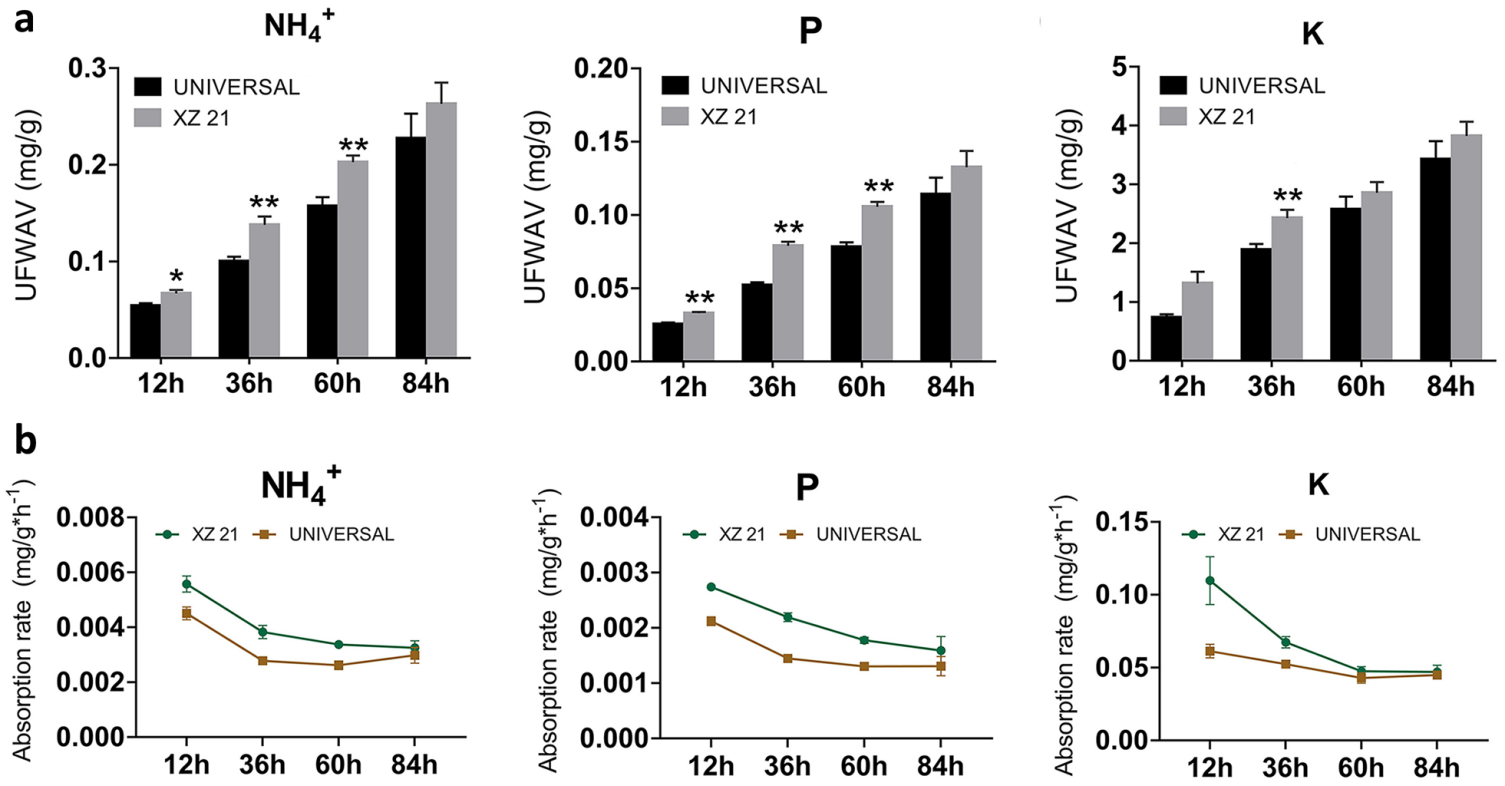
The absorption values (**a**) and absorption rates (**b**) of various elements in wheat seedlings under different treatment times. The absorption rate of wheat seedlings was indicated by the absorption value of per unit fresh weight per hour. UFWAV: unit fresh weight absorption value. Values are means of at last six replications  ±  SE. *t test significant difference. *P  <  0.05, **P  <  0.01

To evaluate the reliability of this method and determine the optimal treatment time, two wheat cultivars and four treatment times (12 h, 36 h, 60 h, 84 h) were selected for the evaluation. The absorption of the all three elements (NH_4_^+^, P and K) increased with the treatment duration, and showed significant difference in absorption between two tested cultivars (Fig. 2a). Meanwhile, the contents of three elements in the control tube remained constant at any quantification time (Additional file 1: Figure S1), indicating the mineral elements were absorbed in wheat seedlings, and the quantification procedure is reliable. To determine the optimal treatment time point, the absorption rates of three elements were analyzed (Fig. 2b) and showed that the absorption rate of three elements gradually decreased with the treatment duration, and then plateaued at 60 h. In addition, the absorption of Mg, Ca, Mn and Zn in wheat seedling could also showed a stable value after 60 h treatment (Additional file 1: Figure S2). Therefore, 60 h treatment was selected for subsequent investigations.

### Correlation of absorption of seven mineral elements in wheat seedling

**Fig. 3:**
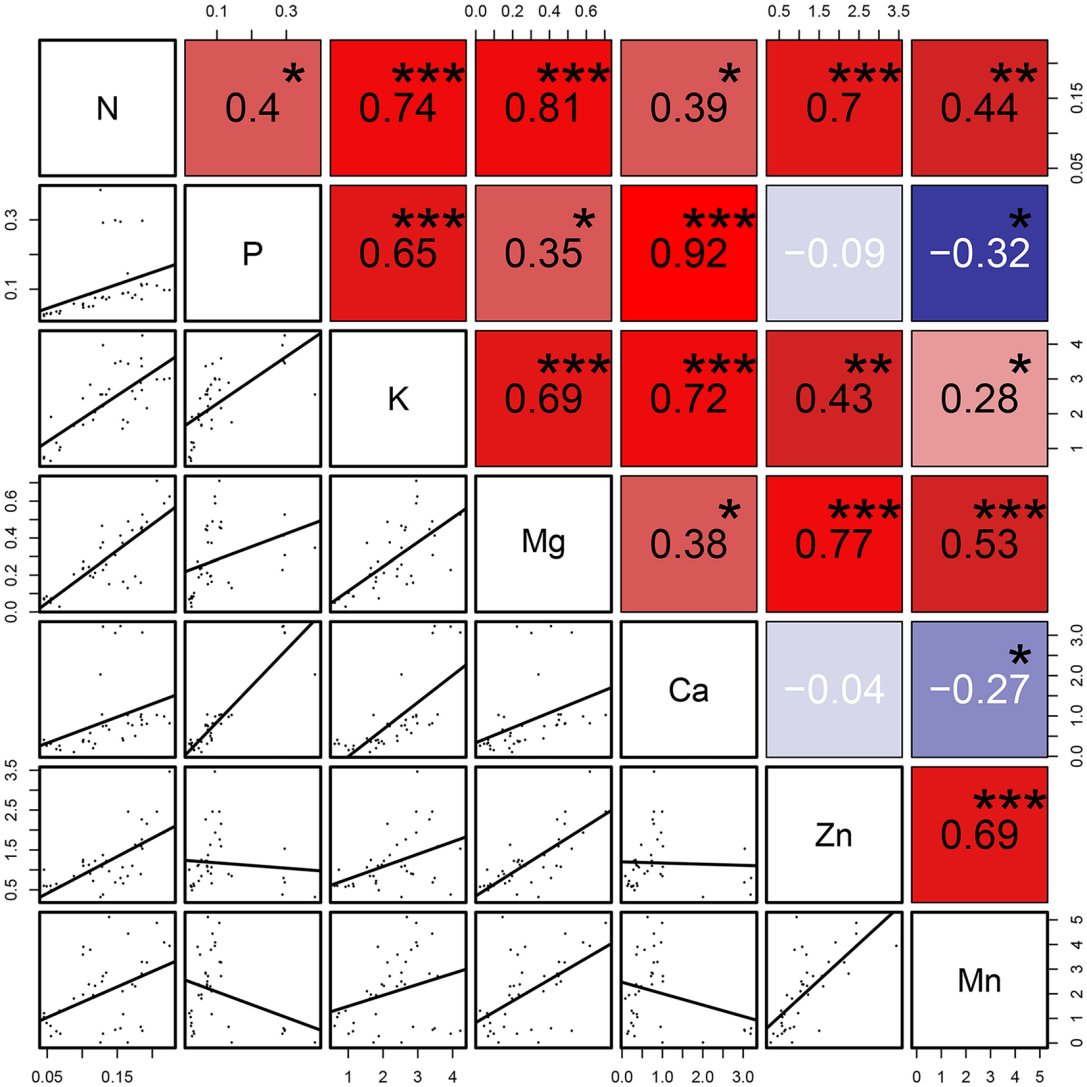
Pearson correlation analysis matrix between elements. *Significant correlation. *P  <  0.05, **P  <  0.01, ***P  <  0.001. The fitting function are shown in Additional file 2: Table S1

To understand the coordinated absorption of different elements in wheat seedling, a correlation analysis was performed (Fig. 3), and showed a significant positive correlation among the five macronutrients (N, P, K, Mg and Ca), consistent with the previous reports [30, 31]. A new finding is that trace element Zn is significantly positive correlated with macronutrients N, K, Mg and trace element Mn, indicating that Zn plays an important role in mediating nutrient absorption of wheat.

### Zn significantly affects the uptake of other mineral elements in wheat seedling

**Fig. 4:**
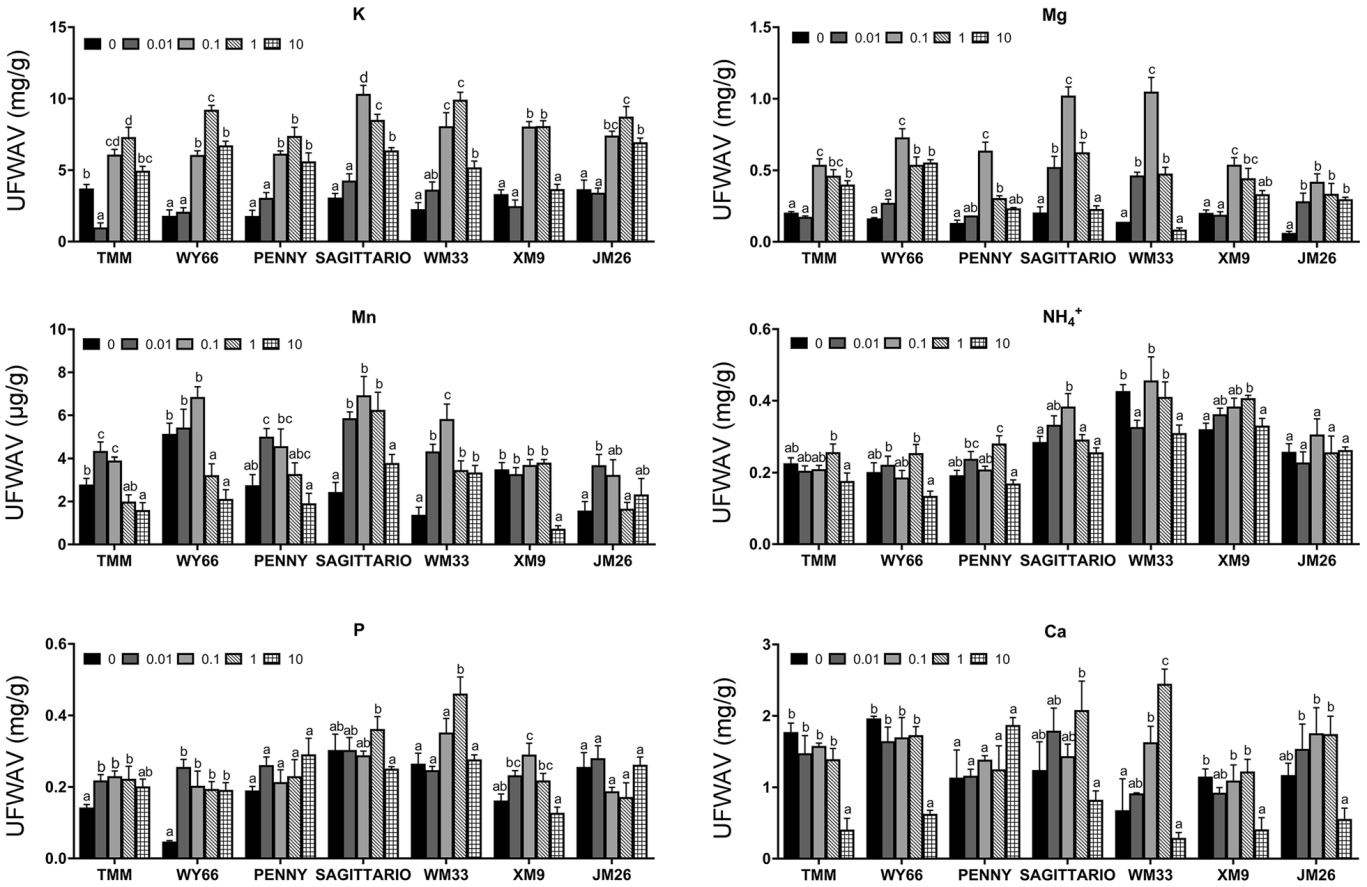
Effects of different Zn concentrations on the absorption of mineral elements in wheat seedlings. UFWAV: unit fresh weight absorption value. Values are means of at least six replications  ±  SE. Within each cultivar, means with different lowercase letters are significantly different at P  <  0.05

To further clarify the role of Zn in mediating mineral elements absorption, five Zn concentrations were performed on seven wheat cultivars. Results showed the uptake of Zn in all seven cultivars is significantly increased with the increase of Zn concentration (Additional file 1: Figure S3). In different Zn concentrations, the absorption of K, Mg and Mn in all seven wheat cultivars is promoted by low concentration Zn (<  1 μM) but is inhibited by the high concentration (10 μM), and suggesting 0.01–0.1 μM Zn in solution have the optimal effect to promote the uptake of other mineral elements (Fig. 4). However, the uptake of NH_4_^+^ and P seems not be affected by different Zn supply (Fig. 4). These results displayed the role of Zn in regulating the absorption of specific mineral elements in wheat seedling.

Interestingly, the uptake of Ca showed distinct response among different cultivars with the alterations of Zn concentration (Fig. 4), indicating the interaction between Zn and Ca uptake is highly variable in different wheat cultivars, which may be determined by the different genetic background of different wheat cultivar.

### The potential role of dwarfing gene *Rhts* in mineral elements uptake

**Fig. 5:**
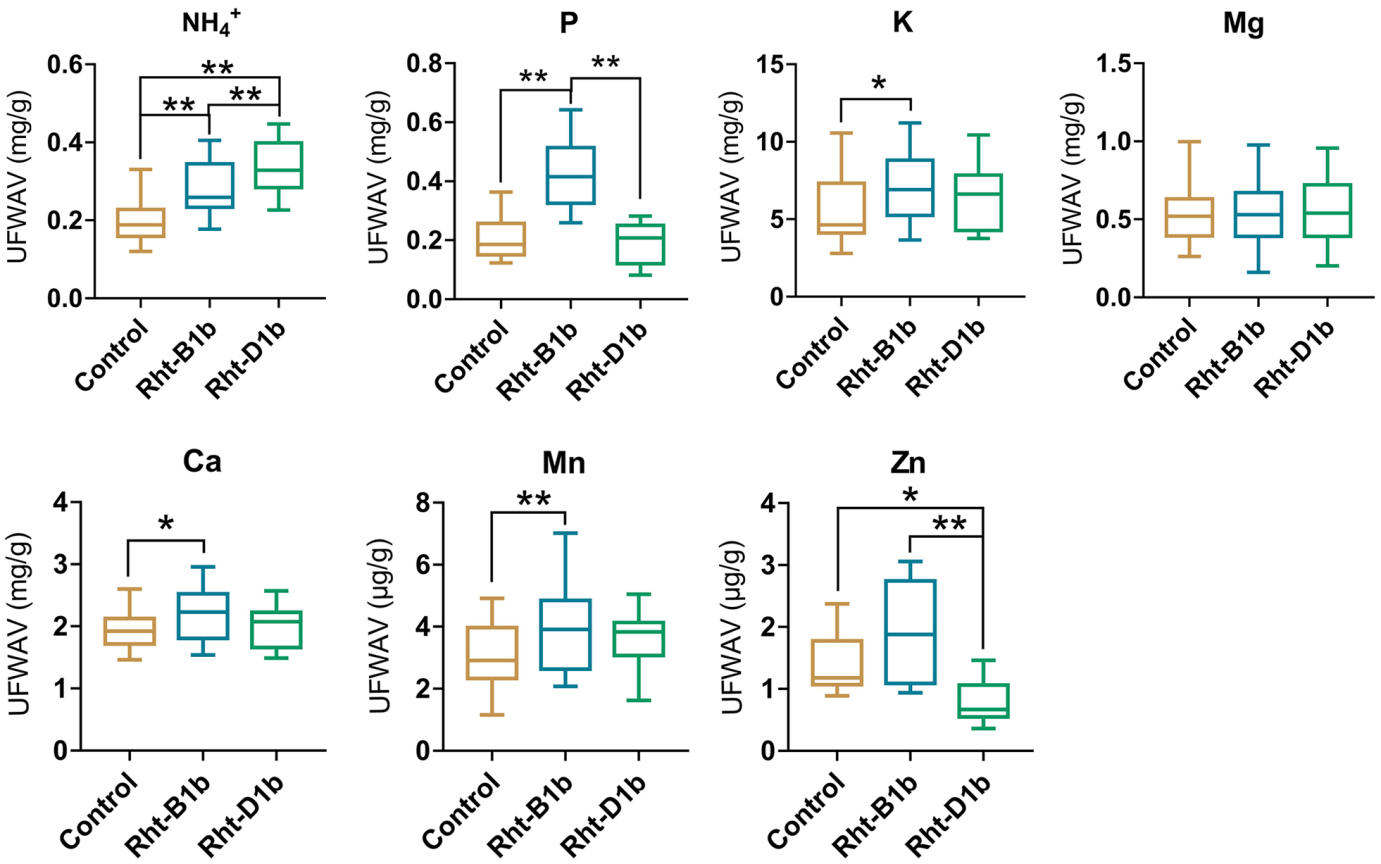
Effects of *Rhts* allele on absorption of mineral elements in wheat. UFWAV: unit fresh weight absorption value. The control group contains seven cultivars, and the *Rht-B1b* and *Rht-D1b* groups respectively include four cultivars, and each cultivar has at least six replicates. Values are means of all replicates of all cultivar in each group  ±  SE. *t test and ANOVA significant difference. *P  <  0.05, **P  <  0.01. The results of ANOVA are shown in Additional file 2: Table S2

Previous studies suggested that the dwarfing gene *Rhts* significantly increases the uptake for nitrogen fertilizer in crops [26, 32]. To further test our method, eight new wheat cultivars were added into our investigation. All fifteen wheat cultivars (see methods) were classified into three groups: *Rht-B1b* allele contained group, *Rht-D1b* allele contained group and Control group (without either *Rht-B1b* or *Rht-D1b* allele). We found the uptake of NH_4_^+^ is significantly increased in both *Rht-B1b* and *Rht-D1b* group (Fig. 5) with medium Zn supply (1 μM), consistent with previous conclusion [23], further supporting the special role of green gene in mineral elements absorption.

In addition, *Rht-B1b* showed a significantly higher uptake of P, K, Ca and Mn compared to control group, and a higher absorption in P and Zn compared to *Rht-D1b* group (Fig. 5). However, neither *Rht-B1b* nor *Rht-D1b* significantly affected Mg absorption (Fig. 5). These results indicate that *Rhts* alleles contribute a broad effect in the absorption of mineral elements in addition to nitrogen, and suggest a different mineral element uptake potential between *Rht-B1b* and *Rht-D1b*.

### The interaction between Zn supply and *Rhts* alleles on uptake of mineral elements

In low Zn concentration (< 1 μM), both *Rht-B1b* and *Rht-D1b* group showed a significantly increased absorption on NH_4_^+^ with the Zn supply increase, and *Rht-B1b* and *Rht-D1b* group showed enhanced absorption of P and Mg, respective (Additional file 1: Figure S4). While the *Rht-D1b* group showed a significantly decreased absorption on Ca and Mn with Zn supply increase (Additional file 1: Figure S4).

**Fig. 6:**
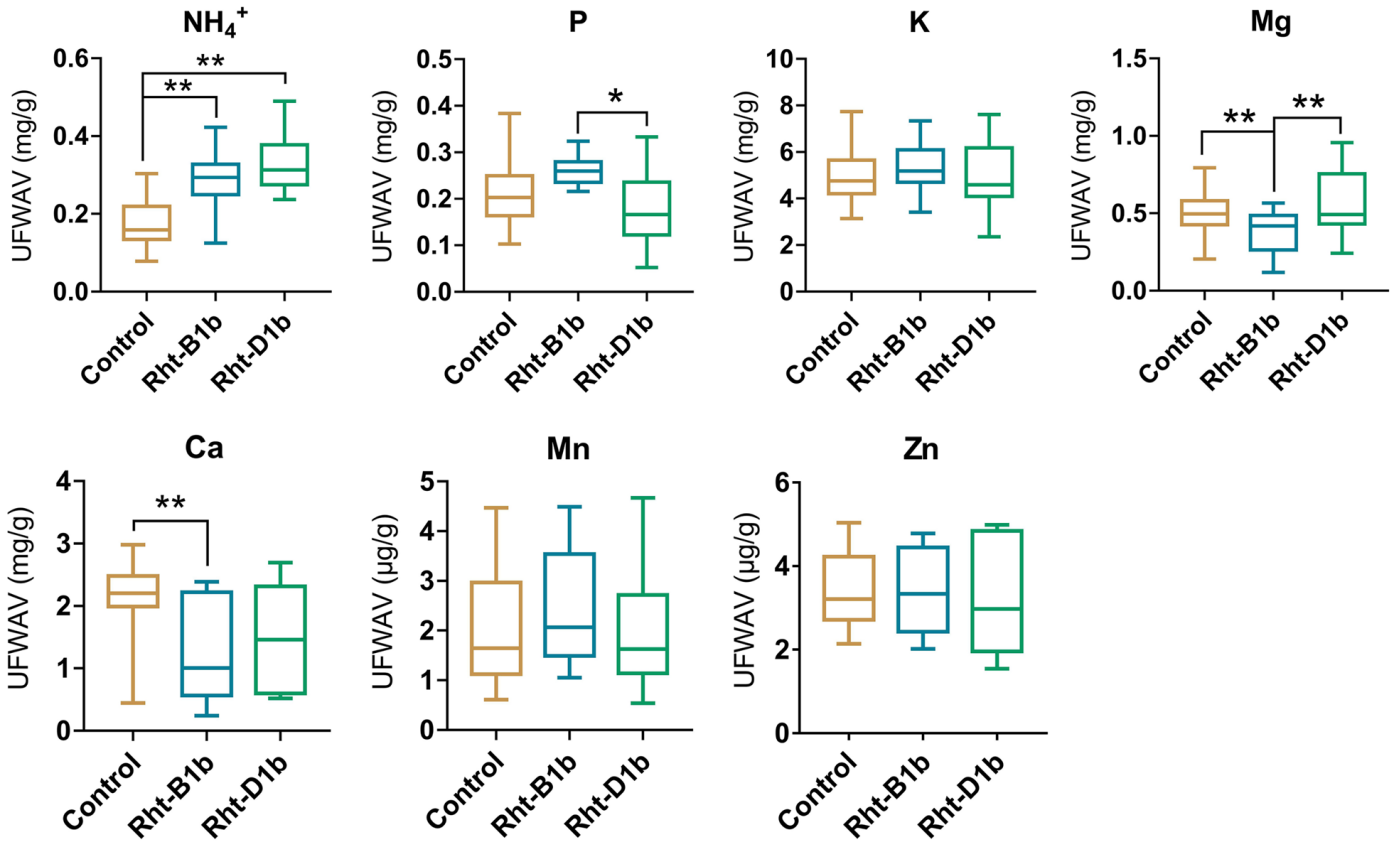
Effects of high Zn concentration (10 μM) on the absorption of mineral elements in wheat containing the *Rhts* allele. UFWAV: unit fresh weight absorption value. The control group contains seven cultivars, and the *Rht-B1b* and *Rht-D1b* groups respectively include four cultivars, and each cultivar has at least six replicates. Values are means of all replicates of all cultivar in each group  ±  SE.*t test and ANOVA significant difference. *P  <  0.05, **P  <  0.01. The results of ANOVA are shown in Additional file 2: Table S2

In high Zn concentration (10 μM), *Rht-B1b* and *Rht-D1b* group still maintained the higher absorption on NH_4_^+^ compared control group (Fig. 6), indicating that the higher NH_4_^+^ absorption is independent of Zn concentration. Similarly, the absorption of P, K, Ca, Mg, Mn was not significantly inhibited by high Zn concentration in *Rht-D1b* group (Fig. 6), compared to the absorption in medium Zn concentration (Fig. 5). In contrast, the absorption of these elements was significantly inhibited in *Rht-B1b* and control group, highlighting a special interaction between Zn and *Rht-D1b* in mineral elements absorption.

## Discussion

In this study, we developed a new method to quantify the absorption of mineral elements in wheat seedling. Our data with this method demonstrated highly stable and reproducible, indicating it is a robust method, which could be applied to study the role of different elements in wheat nutrient coordinated absorption, and may be extend to other crops.

Although its robustness, there are still some disadvantages that should not be overlooked. Firstly, the NO_3_^−^ ions, another important nitrogen form [33], failed to be quantified in this method. Secondly, few trace elements cannot be effectively detected due to their low content nature. Thirdly, the soil environments and related microorganism context played the core role in determining the absorption of soil mineral elements [34, 35], thus this method definitely need further evaluate and improve to reflect the real status of nutrients uptake by crop in field condition. Then based on this initial method here, it is promised to develop a comprehensive and precise method for futural study in plant nutrients absorption.

In this study, our results (Fig. 4) indicate that Zn broadly affects K, Mg, and Mn uptake, supporting low concentration of Zn limited the absorption of K and Mg [12, 36] and high concentration of Zn significantly inhibits the absorption of Mn and Ca [37–39]. This result further confirms that excessive Zn caused a negative interferences to others nutrients uptake [16]. It should be noted that the absorption of NH_4_^+^ in different Zn concentrations keeps stable in this study (Fig. 4d), which is different with the positive interaction relationship between Zn and nitrogen in previous report [40]. We assume the difference may result from the quantification on uptake of nitrate is missed in our study, but this hypothesis needs further comprehensive investigation. Importantly, a general optimum concentration of Zn was proposed, the 0.01–0.1 μM of Zn in solution can promote the uptake of most mineral elements, providing a reference concentration for futural Zn fertilizer application.

Consistent with the previous study [23], both *Rht-B1b* and *Rht-D1b* significantly increased NH_4_^+^ uptake by wheat seedling. Moreover, in our results *Rht-B1b* and *Rht-D1b* contribute a distinct influence in the uptake of mineral elements, and distinct response to alteration of Zn concentrations. Although limited wheat cultivars were investigated in this study, then these distinct responses between the *Rht-B1b* and *Rht-D1b* implying that our method may provide important clues for studying the effect of different genes on the mineral element absorption in plant.

## Conclusions

In summary, the new method developed in this study have a robust capability to investigate the coordinated mineral elements absorption in wheat seedling, and promisingly extend to other crops. With this method, we identified the key role of Zn in coordinated absorption of mineral elements in wheat seedling, which provides a reference to properly apply Zn for enhancing wheat production.

## Methods

### Plant materials

In this study, according to whether it contained *Rht-B1b* and *Rht-D1b* alleles, fifteen wheat cultivars were used and divided into three groups: Control group: UNIVERSAL, Chuannong12 (CN12), Nanda96co76 (ND96), GHARFLOR1611 (G1611), Tumangmai (TMM), Wanyuan66 (WY66), PENNY; *Rht-B1b* group: Xuzhou21 (XZ21), Xiaoyan22 (XY22), SAGITTARIO, Wanmai33 (WM33) and *Rht-D1b* group: Aikang58 (AK58), Zhongmai9 (ZM9), Xinmai9 (XM9), Jimai26 (JM26).

### Plant culture and treatments

Seeds of the wheat were surface-disinfected in 5% sodium hypochlorite for 10 minutes, then rinsed in distilled water for six times, and soaked in a low-temperature (4 °C) incubator for vernalization 3 days. The vernalized seeds were transferred into germinating box (15  ×  15 cm, 16 seedlings per box) with filter paper and cultured in sterile distilled water. In addition, replacing 15 ml distilled water every two days. During this period, no mineral nutrients were added. These seedlings were cultured in a growth chamber with 22 °C/18 °C (day/night), 16 h/8 h (light/dark, light intensity, 2000 lx), and 50% humidity.

After 14 days of germination, well-grown wheat seedlings from each cultivar were selected and transferred into a test tube containing 1/2 Hoagland nutrient solution (Fig. 1). The composition of the nutrient solution was as follows: 2 mM Ca(NO_3_)_2_·4H_2_O, 3 mM KNO_3_, 1 mM MgSO_4_·7H_2_O, 10 μM Fe-EDTA, 0.5 mM NH_4_H_2_PO_4_, 23 μM HBO_3_, 1 μM ZnSO_4_·7H_2_O, 0.15 μM, MnCl_2_·4H_2_O, CuSO_4_·5H_2_O and 0.05 μM Na_2_MoO_4_·2H_2_O. Notably, to avoid failure detection of trace elements, two seedlings were put into each test tube. In addition, the opening of the tube is sealed with plastic film to prevent the evaporation of nutrient solution. Then, these seedlings were transferred to the growth chamber with the same growth parameters for different treatment time.

### Time gradient treatment

Two cultivars were selected for treatment at four time points (12 h, 36 h, 60 h, 84 h). The treatment time is calculated from the time when the seedlings are placed in a test tube containing nutrient solution to the time when they are removed. At least 6 replicates were performed for each cultivar under each time treatment, with one test tube as a replicate. In addition, test tubes without treatment of seedlings were used as controls and at least 6 replicates were also performed under each time treatment.

### Zn concentration gradient treatment

In this study, five Zn concentration gradients of 0, 0.01, 0.1, 1, 10 μM were selected. Zn concentration was changed by changing the content of ZnSO_4_·7H_2_O on the basis of 1/2 Hoagland nutrient solution, while the content of other elements remained unchanged. In addition, all treatments and culture conditions were consistent with those described above.

### Determination of absorption value of mineral elements

After treatment, the wheat seedlings were taken out of the test tubes, and the fresh weight of seedlings and the volume of remaining nutrient solution were measured. Next, prepare standard solutions for each element, and then use Flame/Graphite Furnace Atomic Absorption Spectrometer “Z2000” (Hitachi, Japan) and continuous flow analyzer “FLOWSYS” (Systea, Italy) to detect the absorbance value of each element, and finally obtain the standard solution curve and curve equation of each element. Then detect the absorbance value of each element in the test tube of the control group and the treatment group, and calculate the concentration of each element through the curve equation.

Note: the absorbance value of total Zn, Mn, Cu, Mg, K and Ca in the nutrient solution in the test tube were measured by Atomic Absorption Spectrometer. Due to the limited detection range of the instrument, the nutrient solution must be diluted 10 times to measure the concentration of Mg, and diluted 100 times to measure the concentration of K and Ca. The absorbance value of total NH_4_^+^ and P in the nutrient solution were measured by continuous flow analyzer, in which the measurement of element P requires dilution of the nutrient solution by 5 times. The concentrations of all elements in the nutrient solution of the untreated seedlings were as the control.

In order to avoid the influence of the different growth status of wheat seedlings on the measurement results, the absorption value per unit fresh weight was used as the index to evaluate the nutrient absorption capacity of wheat in this study. Unit fresh weight absorption value (UFWAV)  =  (control measurement value × volume − sample measurement value × volume)/fresh weight.

### Statistical analysis

Use the software SPSS (IBM SPSS Statistics 23.0) for statistical analysis of the data, and use the analysis of variance (ANOVA) for the significance of each treatment effect and its interaction. When the results of the analysis of variance show a significant difference, the Tukey’s HSD test is used to determine the significant difference between the means (P  <  0.05).

## Supplementary Information

**Additional file 1: Figure S1.** The concentration of each element in the control group under different treatment time. **Figure S2.** The unit fresh weight absorption values of Mg, Ca, Mn and Zn in two wheat cultivars after 60h treatment. **Figure S3.** Zn absorption values of seven cultivars treated with different Zn concentrations (1 μM and 10 μM). **Figure S4.** Effects of low Zn concentration (0–0.1 μM) on the absorption of mineral elements in wheat containing the *Rhts* allele.

**Additional file 2: Table S1.** Fitting function of Pearson correlation analysis between elements. **Table S2.** Variance analysis of absorption values of various elements for different group cultivar.

## Data Availability

Not applicable.
