# Supplementary material for: Meta-analysis of the role of zinc in coordinating absorption of mineral elements in wheat seedlings
Source: Plant Methods. 2021 Oct 12;17:105. doi: 10.1186/s13007-021-00805-7 (PMC8513278; doi:10.1186/s13007-021-00805-7)
Supplement: Supplementary file 1 — Additional file 1: Figure S1. The concentration of each element in the control group under different treatment time. Figure S2. The unit fresh weight absorption values of Mg, Ca, Mn and Zn in two wheat cultivars after 60h treatment. Figure S3. Zn absorption values of seven cultivars treated with different Zn concentrations (1 μM and 10 μM). Figure S4. Effects of low Zn concentration (0–0.1 μM) on the absorption of mineral elements in wheat containing the Rhts allele. [file 13007_2021_805_MOESM1_ESM.docx]

**Additional file 1:**


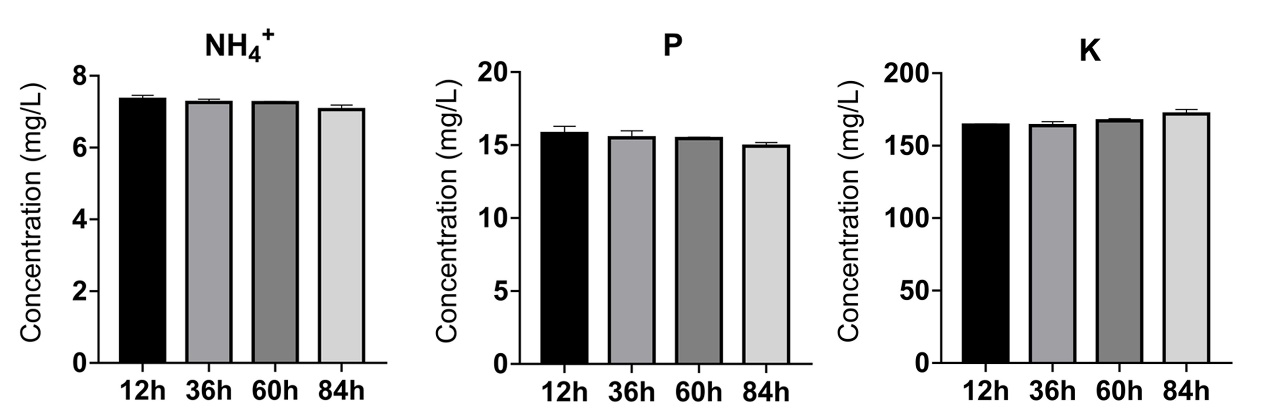


**Figure S1.** The concentration of each element in the control group under different treatment time. Values are means of at last six replications ± SE

**
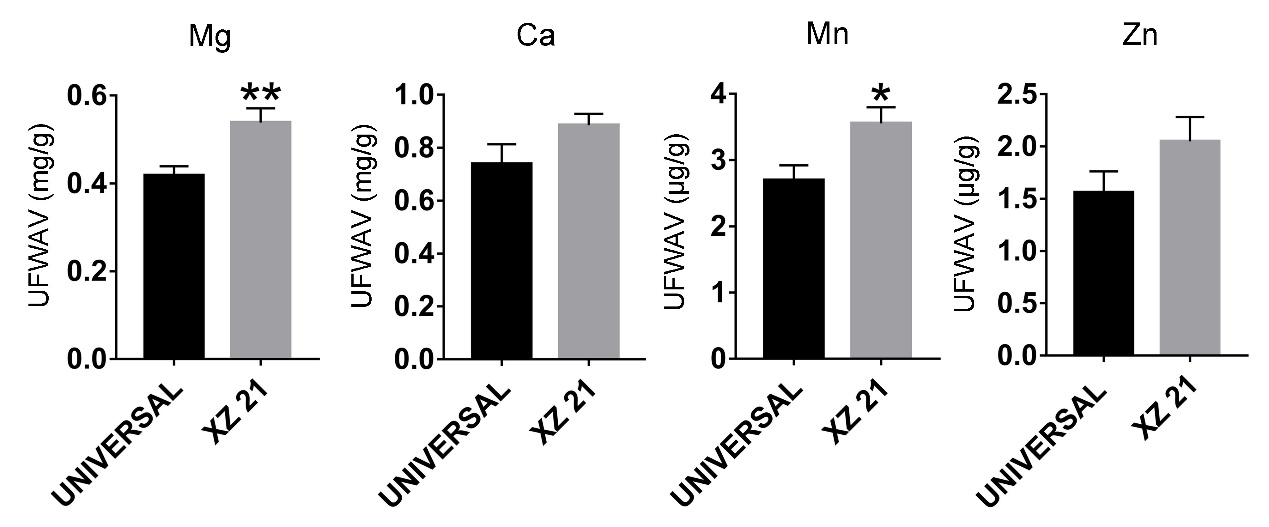
**

**Figure S2.** The unit fresh weight absorption values of Mg, Ca, Mn and Zn in two wheat cultivars after 60h treatment. UFWAV: unit fresh weight absorption value. Values are means of at last six replications ± SE. * Means t-test significant difference * P< 0.05 ** P<0.01

**
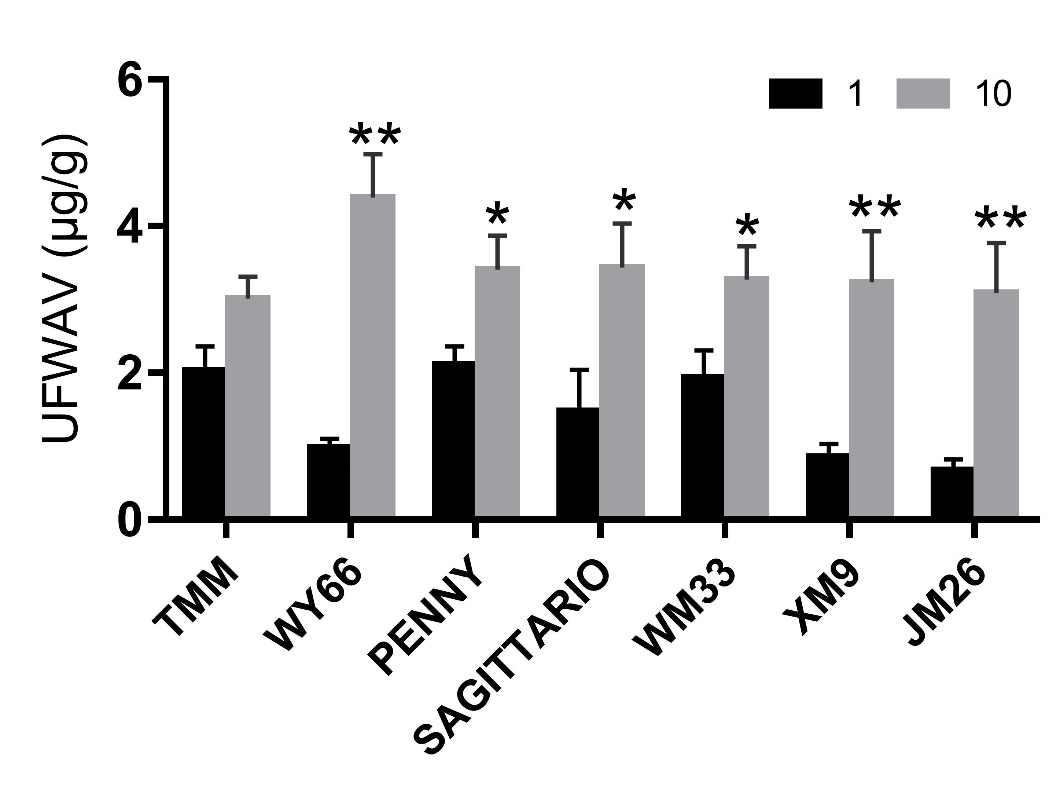
**

**Fig. S3** Zn absorption values of seven cultivars treated with different Zn concentrations (1μM and 10μM). UFWAV: unit fresh weight absorption value. Values are means of at last six replications ± SE. * Means t-test significant difference * P< 0.05 ** P<0.01

**
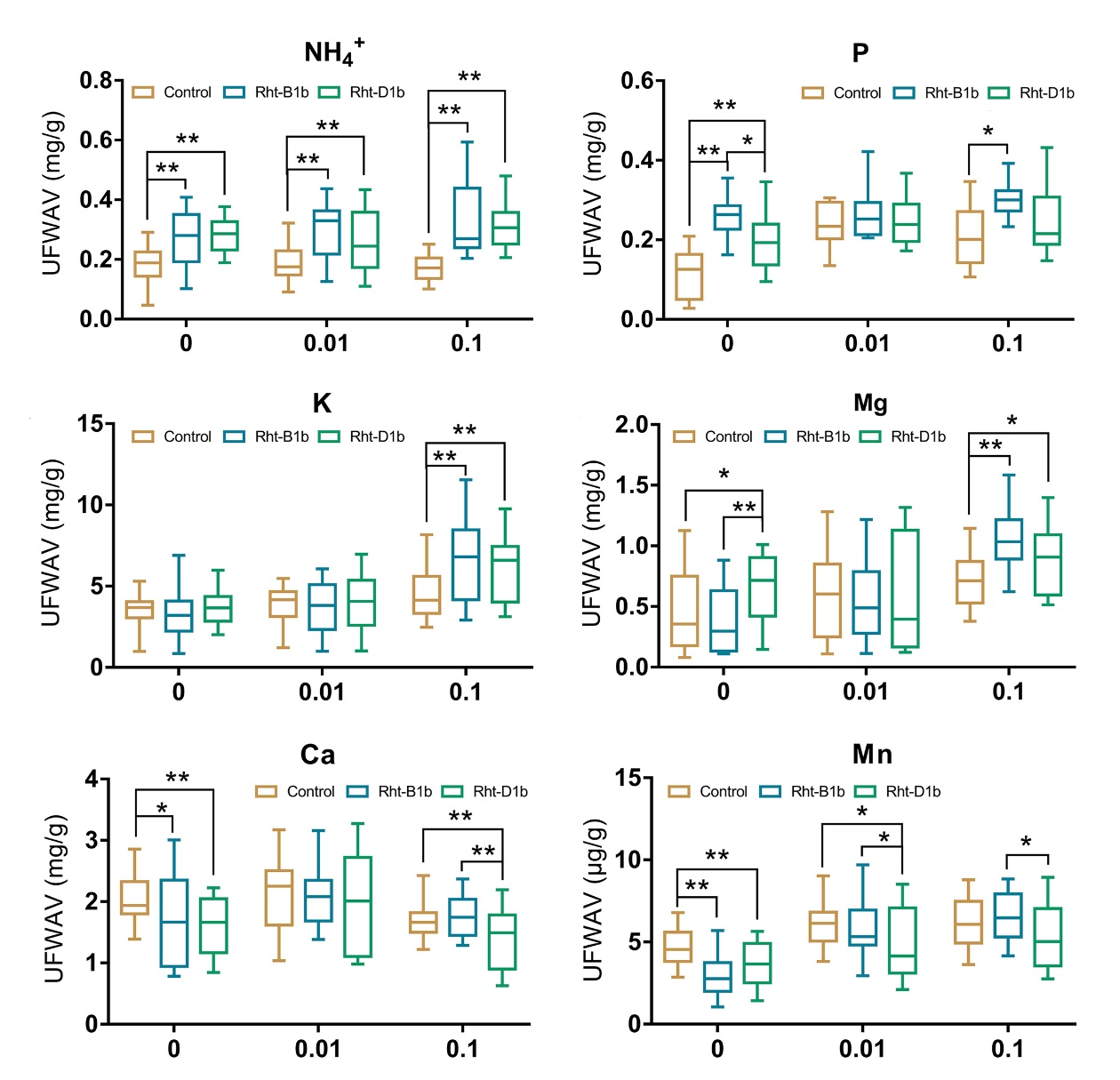
**

**Fig. S4** Effects of low concentration (0-0.1μM) Zn on the absorption of mineral elements in wheat containing the *Rhts* allele. UFWAV: unit fresh weight absorption value. The wild type group contains seven cultivars, and the *Rht-B1b* and *Rht-D1b* groups respectively include four cultivars, and each cultivar has at least six replicates. Values are means of at all replicates of all cultivar in each group ± SE. * Means t-test and ANVOA significant difference * P< 0.05 ** P<0.01. The results of ANOVA are shown in Additional file 2: Table S2.
